# State-of-the-Art Technology of Model Organisms for Current Human Medicine

**DOI:** 10.3390/diagnostics10060392

**Published:** 2020-06-10

**Authors:** Masamitsu Konno, Ayumu Asai, Toru Kitagawa, Masami Yabumoto, Ken Ofusa, Takahiro Arai, Takaaki Hirotsu, Yuichiro Doki, Hidetoshi Eguchi, Hideshi Ishii

**Affiliations:** 1 Center of Medical Innovation and Translational Research, Osaka University Graduate School of Medicine, Suita, Yamadaoka 2-2, Osaka 565-0871, Japan; mkonno@cfs.med.osaka-u.ac.jp (M.K.); aasai@cfs.med.osaka-u.ac.jp (A.A.); toru@kyowakai.com (T.K.); yabumoto.masami@gmail.com (M.Y.); oof21443@ideacon.co.jp (K.O.); t.arai@unitech-op.com (T.A.); hirotsu@hbio.jp (T.H.); ydoki@gesurg.med.osaka-u.ac.jp (Y.D.); heguchi@gesurg.med.osaka-u.ac.jp (H.E.); 2 Artificial Intelligence Research Center, The Institute of Scientific and Industrial Research, Osaka University, 8-1 Mihogaoka, Ibaraki, Osaka 567-0047, Japan; 3 Department of Gastroenterological Surgery, Graduate School of Medicine, Osaka University, Suita 565-0871, Japan; 4 Kyowa-kai Medical Corporation, Hyogo 666-0015, Japan; 5 Kinshu-kai Medical Corporation, Osaka 558-0041, Japan; 6 Prophoenix Division, Food and Life-Science Laboratory, Idea Consultants, Inc., Osaka-city, Osaka 559-8519, Japan; 7 Unitech Co., Ltd., Kashiwa 277-0005, Japan; 8 Hirotsu Bio Science Inc., Tokyo 107-0062, Japan

**Keywords:** biology, diagnosis, therapy, regenerative medicine

## Abstract

Since the 1980s, molecular biology has been used to investigate medical field mechanisms that still require the use of crude biological materials in order to achieve their necessary goals. Transcription factor-induced pluripotent stem cells are used in regenerative medicine to screen drugs and to support lost tissues. However, these cells insufficiently reconstruct whole organs and require various intact cells, such as damaged livers and diabetic pancreases. For efficient gene transfer in medical use, virally mediated gene transfers are used, although immunogenic issues are investigated. To obtain efficient detective and diagnostic power in intractable diseases, biological tools such as roundworms and zebrafish have been found to be useful for high-throughput screening (HST) and diagnosis. Taken together, this biological approach will help to fill the gaps between medical needs and novel innovations in the field of medicine.

## 1. Introduction

The background of morphology and physiology was established in the Renaissance era between the 14th and 16th centuries [1]. In the 19th century, the cell theory of Schwan and Schleiden, i.e., the theory that the cell is the basic unit of every living thing, was proposed in 1838 and 1839 [2]. In the late 19th century, the field of microscopic histopathology was established and was made concrete by Virchow, and Wilson Leopards established microscopic histopathology [3]. Since developments in molecular biology allowed for the research of DNA, RNA, and protein were mainly made in the 1980s [4], this form of modern medicine was constructed on an old foundation, with molecular biology being applied to medicine. One of the characteristics of current medicine was established based on cellular and molecular biology. Recently, biology has been merged with technology in an interdisciplinary manner, increasing the usefulness of the platform. For example, the micro-engineered organ-on-a-chip [5] and the microfluidic organ or body-on-a-chip [6] have been applied in research, diagnosis, and therapeutic approaches, although the ability to uncover uncharacterized mechanisms and to achieve performance, such as sensitivity and specificity, is limited.

**Figure 1:**
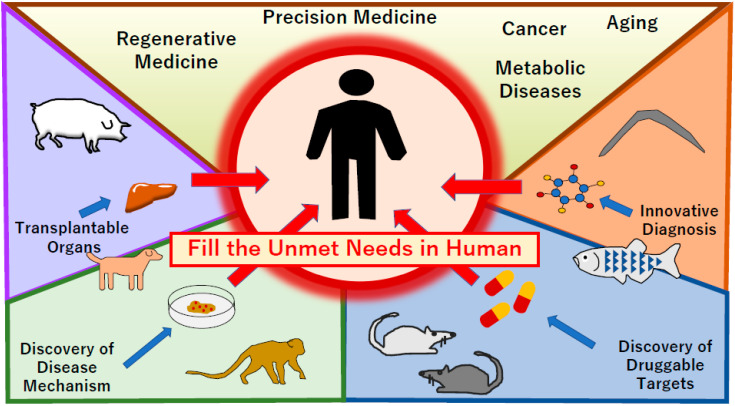
Animal model technologies fulfill the gaps between the present achievement and unmet medical needs in humans. Regenerative medicine needs animal tissue materials such as porcine for the supplementation of organ function in humans (**left**). For the discovery of disease mechanisms, the use of animals such as canines and primates has progressed (**left bottom**). Moreover, disease specific induced pluripotent stem (iPS) cells have been developed. To discover druggable targets, rodent and zebrafish models have been used for the facilitation of drug screening in vivo (**right bottom**). To achieve innovation in the diagnosis of rare diseases and early detection of deleterious conditions, several animal models, including worms, have been applied (**right**). These model systems have contributed to filling unmet needs.

**Figure 2:**
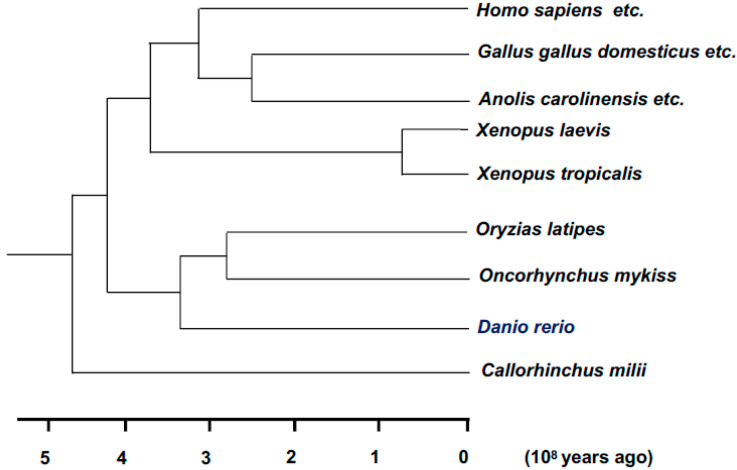
Phylogenetic tree for Homo sapiens (humans) and other model animals. The phylogenetic tree shows that humans and another model animal, Danio rerio, diverged approximately 420 million years ago. However, 82% of human disease-related genes are covered by Danio rerio homologous genes [13]. The figure depicts the phylogenetic tree over the years (per 10^8^ years).

To fill the gap between the current status and unmet needs in medicine, functional drug discovery and diagnosis are critical in the field of drug discovery. This can be achieved through the high-throughput screening (HTS), phenotype screening, and virtual screening of precision medicine, such as in human immunodeficiency infection using bacteria [7], seizures in zebrafish [8], and Duchenne muscular dystrophy in animals such as worms, fruit flies, and zebrafish [9]. This is also important in the fields of detection and diagnosis, such as the use of organic and metal complex-based fluorescent sensing nanoprobes to monitor the level of nitric oxide, an important gaseous signaling molecule related to various human diseases, by using animals, such as mice and zebrafish, in vitro and in vivo (Figure 1 and Figure 2) [10,11,12].

## 2. Regenerative Medicine

Since the discovery of transcription factor-induced pluripotent stem (iPS) technology [14,15], regenerative medicine for corneal endothelial cells [16], cardiocytes [17], hepatocytes [18], and drug screening has been established [19]. Remarkably, the present in vitro technology can increase cell sheets or partial tissues, which are far from organs with complex structures formed after natural development and are not induced by embryonal stem (ES) cells in vivo.

As a multi-functional organ, the induction of islets and exocrine tissues in the pancreas from ES/iPS cells remains difficult and presents hurdles. Since the pancreas is composed of two distinct components, the exocrine pancreas and endocrine islets, regenerative medicine can serve as an avenue for each specific purpose [20]. The loss of islet β cells in type 1 diabetes requires insulin replacement, a hormone secreted from β cells in endocrine islets as therapeutic intervention. However, human islets possess limited regenerative ability. Restoring endocrine function is the most promising strategy. This requires transplanting islets from the patient or generating β cells from ES/iPS cells. This, however, has been too difficult to realize [20]. Other approaches include the induction of insulin-producing β cells or β-like cells in vitro and in vivo. Eventually, several methods were developed to generate or reprogram endodermal cells into β cells or β-like cells in vitro and in vivo [21,22].

Importantly, these approaches do not reach the reprogramming level required to generate the whole pancreatic organ, containing exocrine tissues and endocrine islets, and each cell-separating method is required for regenerative medicine. The use of polymers has facilitated the generation of human stem cell-derived β cells in immunocompetent mice, under long-term glycemic control conditions [23], suggesting that an uncharacterized mechanism is involved in the formation of endocrine tissue. At the present level of technology, the supporting materials, substance, and structures were obtained from the living tissues in vivo.

To facilitate the generation of whole organs, recent studies have reported an innovative technology to generate ES/iPS cell-derived organs in genetically modified animals. Reportedly, a rat pancreas was produced using the interspecific blastocyst injection of pluripotent stem cells in mice, the so-called blastocyst complementation method [24]. In the background of mice with knockout allele of the pancreatic and duodenal homeobox 1 (*Pdx1*) gene, a critical transcription factor that determines the fate of pancreatic endocrine and exocrine differentiation, the injected ES/iPS cells increased the insulin levels in the pancreas in vivo [25,26]. Similarly, reproduction of the lungs using the blastocyst complementation method has recently been reported [26]. The blastocyst complementation method has been successful not only in mice but also in pigs, which are genetically closer to humans [27]. The blastocyst complementation method is beneficial because in vivo tissues harbor uncharacterized mechanisms which cannot be narrowed down to a molecular-based characterization. In a study on the uncharacterized mechanisms of hereditary diseases, the blastocyst complementation method was shown to be beneficial. The application of the blastocyst complementation method has also been found to be useful in human hereditary pancreatitis [28]. Thus, the creation of organs using adult animals has been actively studied. The field of regenerative medicine will progress by using these methods.

## 3. Biological Detection and Diagnosis

### Dogs

Humans have several million olfactory cells and dogs have approximately 200 million. By comparison, humans have 350 variations of olfactory receptors and dogs have 870. A recent study on memory tests revealed that dogs were superior to rats and that both dogs and rats were superior to humans [29]. Therefore, animals are better suited to objective testing.

**Figure 3:**
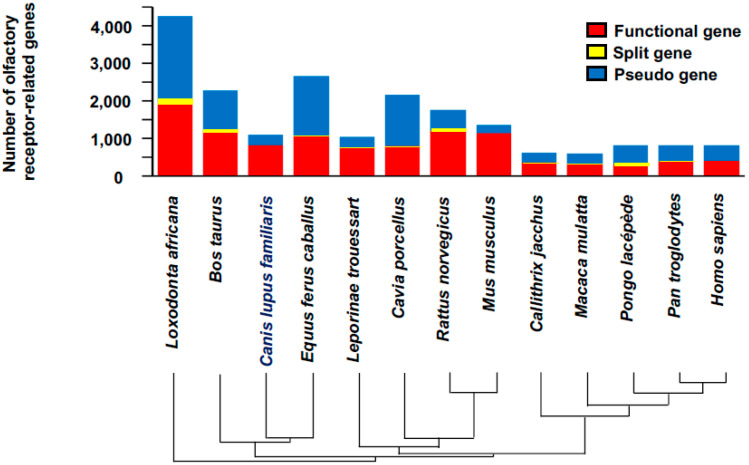
The number of olfactory receptor-related genes. Given that olfactory function can be used for the diagnosis and monitoring of conditions, the study of the number of olfactory receptor-related genes indicates that Homo sapiens posess 396 kinds of functional olfactory receptor-related genes. By contrast, Canis lupus familiaris have 811 types of functional genes, suggesting the rationale that Canis lupus familiaris have a better sense of smell than Homo sapiens [32].

A double-blind procedure was performed in order to examine the ability of beagles to discriminate fresh biopsy and discharge samples from patients with cervical cancer, suggesting that trained dogs may be useful for noninvasive alternative methods to detect cervical cancer in patients [30]. Another study indicated that the canine olfaction can detect liquid samples from breast cancer and colorectal cancer cell cultures, although the dogs were not able to discriminate between the odors of metabolic wastes from breast and colorectal cancers [31]. The cost of training and maintaining the dog is relatively high. In addition, the limited number of times it can be tested means that it cannot be used multiple times a day (the number of olfactory receptor-related genes in other model animals is shown in Figure 3).

## 4. *Caenorhabditis elegans* (*C. elegans*)

*C. elegans* has been used to distinguish patients with cancer from healthy patients by using their sensitivity to smell on substances such as a urine samples [33]. The sensitivity and specificity of this detection ability have been optimized to analyze the chemotaxis or olfactory behavior response alteration, and a kit, known as Nematode-NOSE (N-NOSE) has been developed [34]. Receiver operating characteristic (ROC) analysis indicated that the N-NOSE was higher than that of classical tumor markers. The N-NOSE showed different behaviors before and after tumor removal via surgical operation, suggesting its usefulness as a highly sensitive method to monitor patients postoperatively [34]. The results were confirmed through the analysis of a relatively large study of 180 urine samples from patients with gastrointestinal cancer and 76 samples from healthy participants, indicating the method’s high sensitivity as a gastrointestinal cancer screening test, with a significant value of 0.80 in the receiver operating characteristic analysis, even in early stage cancers [35].

Using the N-NOSE mechanism, the animal experiment conducted on pancreatic cancer demonstrates that *C. elegans* can recognize the odor of pancreatic cancer in the urine of the oncogenic Kras^G12D^ model, whereas the role of c-Met, a receptor of hepatocyte growth factor, was not detected [36], suggesting that the downstream products of Kras^G12D^ may be involved in the alteration of the chemotaxis or olfactory behavior response in *C. elegans.*

## 5. Discovery of Drug Targets Using Animal Models

Model organisms, especially simple animal models, have been used to determine the leading role of new molecular biology in studies, such as determining the full lineage of cell development [37]. However, they are no longer special research materials in biomedicine. Recently, research involving the identification of genes, RNA, proteins, and metabolites in the genome using several simple animal models has become common in biomedical research [38]. Moreover, the method of analyzing a model organism or model animal by comparing it with that of humans has become commonplace in biomedical research [38]. Furthermore, phenotypic screening, which takes advantage of rapid and low-cost precise genome analysis, has become widely used in drug development, antioxidant, antiaging, and healthy life expectancy studies [39].

HTS has been used to screen small-sized molecules in order to interact with molecules in the detection system, which has allowed for the identification of molecular targeting compounds for drug discovery. Given that the clustered regularly interspaced short palindromic repeats (CRISPR)-Cas nuclease system is a powerful tool for genome editing, the CRISPR-based HTS method has been used for approaches to identify functional elements within the noncoding genome [40]. The HTS can be used to identify the modulatory effects of xenobiotics, such as drug-to-drug or drug-to-herb interaction, in enzymes of cytochrome P450, a critical drug-metabolizing enzyme superfamily, which can lead to serious adverse drug reactions or failures [41].

### 5.1. Mus musculus (Mice)

The mouse is the most commonly used model organism among mammals. An advantage of this is that many strains have already been produced, and strains suitable for each study can be selected. Technology for preserving the sperm, eggs, and genital organs must be established [42]. Another advantage is that genetically modified mice can be easily prepared [43]. Conversely, one of its disadvantages is the limited amount of sample materials such as blood. Due to the close genetic background of mice, we can obtain stable experimental results. With the development of genome editing technology, various disease models, such as those of Parkinson’s disease [44], glaucoma [45], and heart disease [46], have been established using mice. Therefore, mouse models will continue to be useful for the development of drug discovery research.

### 5.2. Rattus norvegicus (Rat)

The rat is more convenient for surgical procedures and biological samples than mice. It seems to be more suitable for beginners because it can be easily administered to humans and is warmer than mice. An advantage is that there are established techniques to preserve the sperm, ova, and genital organs, as in mice. However, a drawback of this method is the difficulty to make genetic modifications [47]. This model has an advantage in that data can be easily collated, since rats have so far been used in many physiological experiments. Moreover, a small nerve nucleus such as the brainstem can be analyzed easily using a rat with a large brain. When analyzing neural circuit functions, this model has the advantage that partial brain destruction and local injection of drugs and viruses can be easily administered. Therefore, developing research on the therapeutic target search for brain diseases can progress using rats.

### 5.3. Drosophila melanogaster (Fruit Flies)

The fruit fly is a completely metamorphosing insect that becomes an adult in approximately 10 days after short embryonic, larval, and pupal stages [48]. Since the chromosome of the salivary gland undergoes DNA replication without cell division, polytene chromosomes in the bundled DNA can be observed [48]. Moreover, the homology of genes between the fruit fly and humans is greater than 70%. Therefore, the fruit fly can be used to elucidate the functions of human disease genes and elucidate basic mechanisms of life support [49]. A project to determine the circuit structure by connectomic analysis is also ongoing using fruit flies [50,51]. There has also been an increase in studies that measure neural activity using calcium imaging and patch clamp methods [52]. System neuroscience, such as memory learning and behavior control at the circuit level, can be further developed by combining the above innovative technologies.

### 5.4. Bombyx mori (Silk Moth)

Laboratory animals, especially mammals, are associated with many ethical problems. In particular, silk moths have few ethical problems and are a model organism that has attracted much attention as a substitute for mammals. Silk moths are characterized by their ability to inject blood vessels and the intestinal tract separately. They can also be used for human disease models such as diabetes, hay fever, and infectious diseases [53]. Therefore, silk moths are often considered easy to use for drug discovery.

### 5.5. Schmidtea mediterranea (Flatworms)

The flatworm is a model organism with excellent regenerative abilities. It regenerates no matter how it is cut with each piece becoming a separate flatworm. Cutting the head regenerates the head and cutting the tail because of the concentration gradient of a substance along the longitudinal axis. The flatworm is used as a model organism for regenerative biology [54]. Studies using flatworms can provide a better understanding regarding the totipotency of humans that has not previously been revealed.

### 5.6. Ciona intestinalis (Sea Vase)

The sea vase is used as a model organism to elucidate the life phenomena of chordates. At 18 years after fertilization, tadpole larvae become tadpole-type larvae. Furthermore, the sea vase is easy to handle because it reaches adulthood in a relatively short time period (approximately 3 months) and can be artificially self-fertilized [55]. We are able to track individual cells throughout the development process using the sea vase. Therefore, this organism is considered an attractive experimental animal for single cell analysis.

### 5.7. Strongylocentrotus purpuratus (Sea Urchin)

The sea urchin has long been used as a model organism in developmental biology. Its typical characteristics are that the embryos are transparent and easy to handle, and they are closer to vertebrates than the fruit fly and *C. elegans*, and their genome sequence has already been determined [56]. Large numbers of eggs can be obtained from the sea urchin. Therefore, a large amount of DNA, RNA, and protein can be extracted from embryos at various developmental stages. Thus, it is considered to be very useful for research on congenital diseases.

### 5.8. Xenopus laevis (African Clawed Frog)

The African clawed frog is used to study various problems in developmental biology, such as body axis formation, limb formation, metamorphosis, early development, and meiosis (egg maturation). Moreover, egg extracts prepared from unfertilized eggs have greatly contributed to understanding the molecular mechanisms of cell cycle progression, genomic DNA replication, and distribution [57]. Recently, the whole genome analysis and genome evolution research of the African clawed frog have been completed [58], and the genetic information has become easier to analyze. The African clawed frog will continue to be a very important tool in embryological research and the discovery of drug targets.

### 5.9. Xenopus tropicalis (Western Clawed Frog)

The whole genome has been deciphered for the Western clawed frog and it is attracting attention as a model animal in the post-genome era. It is said to be a good source for research on the complex issues of forward and reverse genetics, the comprehensive analysis of molecular information in living organisms, and the elucidation of endocrine disruption mechanism by chemical substances [59]. Therefore, the Western clawed frog will continue to be an important animal model in the field of embryonic biology and the discovery of drug targets.

### 5.10. Danio rerio (Zebrafish)

Zebrafish have characteristics that make them very suitable for studies, such as a transparent body, transparent egg, in vitro fertilization, in vitro development, and high homology with human genes and tissues [60]. Its advantages include a high fertility rate, short gestation time, and low cost. It is said to be the second best human model organism after mice and rats [61]. This is a model organism that has been used in the field of development and morphogenesis because its eggs are transparent and the fetus can be easily observed in the egg [62]. The zebrafish is one of the few third model organisms that allows in vivo HTS and a completely new paradigm [63]. Although zebrafish drug discovery strategies have not yet reached a global consensus, they offer the first ever in vivo HTS of vertebrates that did not yet exist in the drug discovery process [63]. The zebrafish satisfies the requirements of four domains as a drug discovery model organism [64]. Approximately 82% of human disease-related genes are covered by zebrafish homologous genes [65]. This facilitates the establishment of many human monogenic disease models [65]. Moreover, being a vertebrate is a crucial feature with pathopathological similarity that is often observed in disease-related gene mutations and knockout phenotypes [66]. Conversely, between 1999 to 2008, analysis of a new drug approved by the United States Food and Drug Administration successfully revealed that 62% of innovative drugs were found and established by phenotype screening [67]. To overcome the limitations of the current target-based reverse pharmacology, quantitative phenotype screening is attracting attention, and expectations that the zebrafish will be capable of the quantitative HTS of phenotypes and mechanisms at the biological level are increasing.

### 5.11. C. elegans

For the usage of living models in HTS, according to the steadily increasing number of the methodologies over the years, small molecule screening using *C. elegans* as a model is becoming increasingly well-known [68]. Meta-analysis results from four orthology prediction programs containing 7663 *C. elegans* protein-coding genes matched to 38% of the whole human protein-coding genes, allowing for the generation of a compendium for comparison. This “OrthoList” will be considerably useful for *C. elegans* HTS according to RNAi or CRISPR-Cas screening, by focusing on genes with apparent human orthologs, thus reducing the screening effort by approximately 60% [69]. Accordingly, the use of *C. elegans* can reduce screening costs.

As for the anatomical aspects of this method, *C. elegans* and humans share many corresponding tissues, such as the muscle, the nervous system, and gut. Although the nervous system of *C. elegans* is smaller than that of humans, it uses the most common neurotransmitters found in humans, such as acetylcholine, gamma aminobutyric acid, glutamine, dopamine, and serotonin, which are known to be associated with Parkinson’s disease, schizophrenia, and depression [68]. Previous studies suggested that *C. elegans* can be used as a key model for neurobiological studies on development, differentiation, specification, and synaptic formation [68,70], as well as behaviors such as mating, chemotaxis, feeding [71], and leaning [72]. The use of a living model, *C. elegans*, is an example of a model that falls between the molecular and higher vertebrate becomes.

## 6. Conclusions

In recent years, innovative technology has become increasingly important, and new drug discovery can be achieved via interdisciplinary approaches. Given that the ethical, legal, and social issues are generally higher in clinical studies with human applications than animal experiments, the application of animal models in the field of life science must be emphasized.
